# Different patterns of HIV-1 DNA after therapy discontinuation

**DOI:** 10.1186/1471-2334-5-69

**Published:** 2005-09-12

**Authors:** Maria Carla Re, Francesca Vitone, Laura Sighinolfi, Pasqua Schiavone, Florio Ghinelli, Davide Gibellini

**Affiliations:** 1 Department of Clinical and Experimental Medicine, Section of Microbiology, University of Bologna, Via Massarenti 9-40138 Bologna, Italy; 2 Department of Infectious Diseases, St Anna Hospital, Corso Giovecca, 203-44100 Ferrara, Italy

## Abstract

**Background:**

By persisting in infected cells for a long period of time, proviral HIV-1 DNA can represent an alternative viral marker to RNA viral load during the follow-up of HIV-1 infected individuals. In the present study sequential blood samples of 10 patients under antiretroviral treatment from 1997 with two NRTIs, who refused to continue any antiviral regimen, were analyzed for 16 – 24 weeks to study the possible relationship between DNA and RNA viral load.

**Methods:**

The amount of proviral DNA was quantified by SYBR green real-time PCR in peripheral blood mononuclear cells from a selected group of ten patients with different levels of plasmatic viremia (RNA viral load).

**Results:**

Variable levels of proviral DNA were found without any significant correlation between proviral load and plasma HIV-1 RNA levels. Results obtained showed an increase or a rebound in viral DNA in most patients, suggesting that the absence of therapy reflects an increase and/or a persistence of cells containing viral DNA.

**Conclusion:**

Even though plasma HIV RNA levels remain the basic parameter to monitor the intensity of viral replication, the results obtained seem to indicate that DNA levels could represent an adjunct prognostic marker in monitoring HIV-1 infected subjects.

## Background

Many papers have clearly demonstrated that HIV-1 RNA plasma viral load quantitative determination is a pivotal parameter to monitor viral replication and the effectiveness of HAART therapy [1-5]. In addition, a growing number of observations showed that measurement of HIV-1 DNA proviral load could provide crucial information on the reservoir and dynamics of HIV-1 infection [5,6] since the persistence of HIV-DNA in peripheral blood mononuclear cells (PBMC) and lymph nodes is a major drawback to eradication of infection [7,8]. Quantitative analysis of proviral DNA in HAART-treated patients showed opposite results: on one hand, the decline in DNA load seemed to indicate the long term impact and effectiveness of retroviral treatment [9-12], on the other DNA levels remained stable over several years in PI ART naïve patients [2,13].

Recent studies also indicate that viral replication persists even in individuals with prolonged suppression of plasma HIV-1 RNA levels to fewer than 50 copies/ml [5,8,14-16], confirming that "undetectable viremia" cannot be considered evidence of complete viral replication suppression. The findings of a slow and/or incomplete decay imply that current HAART regimens do not completely suppress viral replication. However the decreasing morbidity and mortality in HAART-treated HIV-1 seropositive patients and the following restoration, preservation of immunologic function and improvement in quality of life demand the ongoing use of these drugs. The new therapeutic challenge is to find new immunological or pharmacological approaches aimed at purging HIV-1 DNA proviral reservoirs [19]. Several recent studies have addressed structured treatment interruption (STI), as conceivable strategy to stimulate and enhance the immune system HIV-1 specific response to tackle viral replication in the absence of chemotherapy [17-19] even though several reports showed that only 10–20% of chronically infected patients achieved a short-term suppression of viral replication [20-22].

Since a growing number of studies involving quantification of cellular HIV-1 DNA acknowledge the importance of accurate quantification of proviral DNA in peripheral blood cells for monitoring diseases progression, we selected a small but peculiar group of patients, who decided to interrupt antiretroviral therapy, irrespective of current guidelines [23] and despite virologic failure. In particular, sequential blood samples of 10 patients under antiretroviral treatment from 1997 with two NRTIs, who refused to continue any antiviral regimen, were analyzed for 16 – 24 weeks to study the possible relationship between DNA and RNA viral load.

## Methods

### Patients

Ten HIV-1 infected adults under antiretroviral treatment since 1997 with two NRTIs (stavudine [D4T] and lamivudine [3TC] or zidovudine [AZT] and lamivudine [3TC] or zidovudine [AZT] and zalcitabina [DDC] or zidovudine [AZT] and didanosine [DDI]) were selected for this study. All these patients refused to continue any antiviral regimen despite an assessed virologic failure (HIV-1 RNA viral load > 50 copies/ml) and were followed-up monthly for a variable period ranging from 16 to 24 weeks up to the moment in which they agreed to begin a new therapeutic protocol. Sequential blood samples were obtained at baseline (time 0: voluntary therapy interruption) and every four weeks (time 1-time 7) and analyzed for viral load (RNA and DNA), and CD4 levels. The baseline characteristics of the patients included in the study are shown in Table 1. All the subjects were enrolled after informed consent according the Helsinki declaration of 1975.

**Table 1:** Baseline characteristics of HIV infected patients enrolled in the study at time of therapy suspension.

*Characteristic*	
Gender	7 males, 3 females
Heterosexual	8
IVDUs	2
Age (mean years ± SD)	36.27 ± 8.32
CD4 count cells × 10^6 ^per l (median)	548.45 ± 63 cells/mmc.
Plasma HIV-1 RNA copies/ml (median)	3.7 × 10^3^

### HIV-1 RNA quantification

All the whole blood samples were centrifuged at 2500 rpm for 20 min and plasma was stored at -80°C until use. Plasma was analyzed for HIV-1 RNA viral using the Quantiplex HIV-RNA-3.0 assay (Chiron Corporation, Emeryville, CA, USA), according to the manufacturer's instructions. The amount of HIV RNA levels was expressed as copy number per ml of plasma and the lowest detection limit of the assay was 50 copies/ml.

### DNA extraction and purification of PBMCs for HIV-1 DNA quantification

Peripheral blood mononuclear cells (PBMC) were isolated from whole blood by Ficoll-Paque gradient separation (Amersham Pharmacia). Cell pellets, corresponding to 5 × 10^6 ^PBMC were prepared and stored at -80°C. DNA was extracted and purified from each PBMC pellet by DNAeasy tissue kit (Qiagen) following the manufacturers' instructions. The pGEMBH10 HIV plasmid [8,14] was purified by Midi plasmid extraction kit (Qiagen, Hilden, Germany) following the manufacturers' instructions. Plasmid and cellular DNA concentration and purity were determined by spectrophotometric analysis at 260/280 nm.

### Determination of HIV-1 proviral DNA by SYBR green real-time PCR

SYBR green real-time PCR assay was performed, as previously described [8,14] in 20 μl PCR mixture volume consisting in 2× Quantitect SYBR Green PCR Master Mix (Qiagen) containing HotStarTaq DNA polymerase, 200 nM of each oligonucleotide primer (SK431, SK462) [24] and 600 ng of DNA extracted from clinical samples (approximately the DNA content of 200.000 cells) or scalar dilution of pGEMBH10 HIV plasmid (from 10^5 ^to 10 copies). Initial activation of HotStar Taq DNA Polymerase at 95°C for 15 min; 45 cycles in four steps: 94°C for 10 s, 60°C for 30 s, 72°C for 30 s, 78°C for 3 s. At the end of amplification cycles, melting temperature analysis was carried out by a slow increase in temperature (0.1°C/s) up to 94°C. Amplification, data acquisition and analysis were carried out by a LightCycler instrument (Roche, Mannheim, Germany) using LightCycler 5.3.2 software (Roche). This software, coupled with the LightCycler instrument, determines the threshold cycle (Ct) representing the number of cycles in which the fluorescence intensity is significantly above the background fluorescence. Ct is directly proportional to log_10 _of the copy number of the input templates with respect to a standard curve generated in parallel. SYBR green molecules bind all double stranded DNA molecules emitting a fluorescent signal, on binding, proportional to the amplicon synthesis during the PCR reaction. This property elicited an accurate analysis of the melting temperature curve of the amplified fragments generated by real-time PCR to determine the detection and quantitation of specific products. Thus the single analysis of fluorescence was performed at 75°C by LightCycler 5.3.2 software in each cycle to rule out any non-specific interference (i.e. dimer primer). All samples from patients were run in duplicate and were also analyzed by SYBR Green real-time PCR for globin gene in a parallel run to check the equal amount in all samples determined by spectrophotometric data as described.

### Statistical analysis

Statistical analysis was carried out using Student's *t*-test or Mann-Whitney test. Correlation was determined by Spearman's rank correlation.

## Results

### Longitudinal analysis of RNA plasma viral load detection by b-DNA assay

As expected, therapy interruption determined a significant increase in RNA viral load in all HIV-1 seropositive patients enrolled in the study. In particular, all patients' plasma showed a significant (Mann-Whitney test p = 0.036) increase in viral load already one month after therapy interruption (time 1) showing a median value of 1.7 × 10^4 ^(4.2 log_10_) in comparison to 3.7 × 10^3 ^copies/ml (3.5 log_10_) observed at median baseline value (time 0). Moreover, plasma viral load reached higher levels [median value of 1,1 × 10^5 ^HIV-1RNA copies/ml (5 log_10_)] at the end of observation period (p = 0.014) (Table 2). Hence, we assessed an increase in viral replication ranging from 0.5 log_10 _to more than 1 log_10 _at the end of observation period (p = 0.00).

**Table 2:** Longitudinal values of HIV RNA and DNA viral load, CD4 levels in patients on long term treatment with two NRTIs from therapy suspension (time 0) onwards.

	RNA Viral Load (copies/ml)	DNA Viral Load (copies/10^6^PMBCs)	CD4 cell count (x10^6^cells/L)		RNA Viral Load (copies/ml)	DNA Viral Load (copies/10^6^PMBCs)	CD4 cell count (x10^6^cells/L)
**Pt 1**				**Pt6**			
Time0	6300		335	Time0	2940		625
Time1	20800	1600	324	Time1	2500	1100	625
Time2	30000	2200	324	Time2	16000	1400	529
Time3	58000	1800	340	Time3	7800	630	625
Time4	100000	2000	300	Time4	25000	620	552
Time5	120000	3800	220	Time5	24000	730	750
				
**Pt2**				Time6	17000	890	483
				
Time0	11000		345	**Pt 7**			
Time1	22000	1200	345	Time0	1600		462
Time2	70000	1900	365	Time1	6800	920	450
Time3	100000	1500	256	Time2	1700	750	456
Time4	150000	1000	304	Time3	4400	910	384
Time5	148000	770	240	Time4	4000	690	380
Time6	170000	990	238	Time5	2700	400	399
				
**Pt3**				Time6	2800	300	342
				
Time0	3600		551	Pt8			
Time1	43000	2300	448	Time0	3970		616
Time2	58100	820	266	Time1	4000	1000	616
Time3	49000	580	560	Time2	85000	660	532
Time4	58000	990	360	Time3	100000	490	361
				
**Pt4**				Time4	84000	230	420
Time0	1220		572	Time5	74000	330	456
Time1	1400	1300	570	Time6	100000	550	460
				
Time2	24000	1890	522	Pt9			
Time3	46000	960	572	Time0	10500		680
Time4	27000	500	576	Time1	14000	1500	624
Time5	32000	700	432	Time2	16000	1000	480
Time6	31000	550	348	Time3	39000	450	588
				
**Pt5**				Time4	33000	1100	520
Time0	4900		494	Time5	145000	3100	390
				
Time1	93000	1500	528	Pt10			
Time2	100000	1050	483	Time0	1130		578
Time3	150000	830	460	Time1	69000	612	420
Time4	250000	1000	404	Time2	66000	1200	320
				Time3	100000	1600	350
				Time4	210000	2800	267

### Longitudinal analysis of PBMC DNA proviral load detection by quantitative real time PCR assay

In parallel experiments, we quantified proviral DNA load in PBMC isolated from patients' whole blood sequential samples at fixed times after therapy suspension. The median number of samples available for each patient was five [1 month after the therapy suspension (time 1) and then each month up to the end of observation period], ranging from two to seven. The median follow-up was 5.5 (4–7) months.

The majority of patients showed a fluctuating trend in DNA viral load. Three patients (N°1, N°9 and 10) showed an increase in DNA viral load detectable from the first through to the last available sample. Even though DNA amount reached a significantly (considered as a variation of 0.5 – 1 log_10_) higher value only in samples from patients N°1 and N°10 (from 3.2 log_10 _to 3.7 log_10 _and from 2.7 log_10 _to 3.4 log_10 _respectively), sequential PBMC samples obtained from patient N°9 exhibited a clear tendency to increase (and from 3.1 log_10 _to 3.5 log_10 _copy of HIV-1 DNA per 10^6 ^PBMCs respectively) redoubling the DNA content. Moreover, most of the other samples obtained from patients N°2, N°3, N°4, N°5 and N°6 showed a swinging course. After an apparent decline in proviral DNA content during the follow-up, in the latest samples a moderate increase in proviral DNA load was observed in PBMC from all patients. In contrast, a decrease of HIV-1 proviral DNA content was noticed from a baseline value of 1.3 × 10^3 ^copies per ml (3.1 log_10_) to 5.5 × 10^2 ^HIV-1 DNA copies per ml (2.7 log_10_) and from 9.2 × 10^2 ^(2.9 log_10_) to 3.0 × 10^2 ^(2.4 log_10_) in patients N°4 and 7 only.

As expected, a statistical analysis of PBMCs HIV proviral DNA content and plasma RNA viral load of all 10 patients failed to disclose any significant correlation between HIV-1 proviral DNA load and HIV-1 RNA viral load (Mann-Wittney test) confirming our previous data [14].

### CD4 cell count determination

All the patients enrolled in the study showed a CD4 reduction during the follow-up. All patients, except two (Patients N°1 and N°2), interrupted therapy with a level of CD4 cells >400 cells/mmc and, as expected, showed a sharp [(N°1 and N°3 (34% reduction), N°2 (31% reduction), N°4 (39%), N°9 (42%), N°10 (53%)] decrease or a moderate decline [N°5 (18% reduction), N°6 (22% reduction) N°7 and 8 (25% reduction)] at the end of our observation period. No correlation was found (r = 0.5, p > 0.005) between the course of DNA viral load and CD4 levels, but high RNA levels were significantly associated with lower CD4 counts, demonstrating a significant inverse correlation between CD4+ cell counts and HIV-1 RNA levels (p = 0.001).

## Discussion

During recent years, planned therapy interruption has been entertained in specific clinical situations even though the potential role of this choice with respect to the balance between risk of disease progression and potential benefits remains to be elucidated. Our study focused on a peculiar group of patients who voluntary opted to suspend antiretroviral therapy for a variable period of time, ranging from five to seven months, despite of virologic failure. Our follow-up ceased when patients agreed to a new therapeutic protocol.

Our study aimed to evaluate the virologic evolution of these subjects focusing on DNA proviral load course, since accurate quantification of HIV-1 DNA in peripheral blood cells is an important parameter for monitoring disease progression and predicting the clinical outcome of infection [3,27-29]. Several studies, mostly addressed to patients under different therapy protocols, have shown that the evaluation of DNA content may have important implications for understanding the virological response to combination therapy [25,26]. Even thought the plasma HIV-1 RNA load is widely considered a direct indicator of viral replication in infected individuals, the formation, stability and turnover of potentially infectious virus in the HIV-1 DNA proviral pool has important indication for the understanding of HIV pathogenesis [5,6,11]. Moreover, Vitone *et al*. [8] recently demonstrated that the decrease in HIV-1 DNA proviral load is inversely correlated to CD4 level in HIV-1 seropositive patients with a persistently undetectable viremia (HIV-1 RNA viral load).

Current data on course of DNA viral load during infection are inconclusive [1-9], but most studies suggest that HIV-1 DNA proviral quantification is useful to monitor the decay of the HIV reservoir towards disease remission, distinguishing "responder" from "non responder" patients [3,28].

Our results, obtained from patients, therapy-free during the virolgical follow-up, showed a viral rebound, one month after therapy suspension, assessed by plasma RNA values in all patients. The analysis of HIV-1 DNA proviral content displayed a clear increase from the baseline value in three patients, confirming that an active viral replication results in elevated viremia (HIV-1 RNA load) and in an increased number of cells containing viral DNA [27,28]. Also, patients who showed an apparent decrease in DNA copy number during the first step of our follow-up, came to present a rebound of DNA in PBMCs at the end of observation period. These observations might suggest that previous therapy controlled the amount of viral DNA only for a limited period of time and a likely viral rebound, as assessed by an increase in DNA amount, was observed only some months later.

Finally, in contrast with other patients, two subjects showed a clear HIV-1 DNA proviral decrease over time, in the absence of therapy and a steady HIV-1 RNA viral load detectable in plasma samples. In both cases a HIV-1 DNA proviral decline due to a long lasting effect of therapy could be ruled out, since both patients showed high levels of viral replication by increasing value of HIV-1 RNA viral load over time. In an attempt to explain the course of HIV-1 DNA proviral in these subjects, we had to take into consideration that our assay, a SYBR green based real time PCR measures both integrated and unintegrated HIV-1 DNA form on PBMCs. There is evidence that only a fraction of integrated and unintegrated HIV-1 DNA is replication competent [25]. Hence, it is possible that most of the HIV-1 DNA, displayed in our two patients, might be mainly represented by integrated DNA fully capable of initiating HIV replication. Our data are confined to results related to proviral DNA in PBMCs, even if we must consider that viral load is also sustained by lymph node trapped CD4 T cells and other non circulating elements [6] that preserve replication competent virus for long periods. In the absence of therapy, a large number of HIV-1 DNA proviral copies might replicate, as assessed by the HIV-1 RNA viral load increase, leading to a relative decline of cellular DNA. In addition, we cannot exclude a further increase in DNA content in a longer follow-up.

Despite contrasting reports on the meaning of DNA proviral content in HIV-1 seropositive patients [2,5,9-12], our data obtained on closely controlled patients, emphasize the interest of studying DNA proviral content in HIV-1 infected patients. Even though it is impossible to define a proviral DNA threshold for use in clinical practice, several data showed that patients with high proviral DNA levels are more likely to experience virological failure than those with lower proviral DNA loads [11]. Moreover the proviral load probably reflects individual parameters because host genetic factors and response to treatment probably are involved in the constituting the pool of infected cells [30,31]. Although RNA viral load provides important information on viral replication, HIV-1 DNA proviral load can be considered an additional marker to provide crucial information, not only during the follow-up of patients under therapy but also for individuals included in structured therapy interruptions protocols. Data obtained from our patients, who were not part of antiretroviral protocols [23], yield important information on the persisting timing of DNA in PBMCs.

## Conclusion

Only careful evaluation of virological and immunological markers is necessary to fully characterize the course of HIV-1 infection and to provide a more complete laboratory-based assessment of disease progression. However, the availability of a new standardized assay such as DNA proviral load will be important to assess the true extent of virological suppression in patients with non-quantifiable plasma viral loads and to verify the efficacy of new immune-based therapies aimed at purging HIV-1 DNA reservoirs. Although the biological meaning of DNA proviral load in PBMCs is not yet clear, several studies [2,3,6,10] suggest that HIV-1 cellular DNA load may be an indicator of spread of infection whereas the plasma RNA load is indicates active infection [2]. However the qualitative and quantitative evaluation of both plasma HIV RNA genome and HIV-1 proviral DNA might prove crucial to understanding the course of HIV-1 infection.

## Competing interests

The author(s) declare that they have no competing interests.

## Authors' contributions

MCR and DG conceived and designed the study. FS and PV developed the HIV-1 DNA real time and performed all the experimental work. LS and FG provided blood samples and clinical information on the patients enrolled in this study. MCR drafted the manuscript and DG reviewed it. All authors contributed to the final version of manuscript, read and approved it.

## Pre-publication history

The pre-publication history for this paper can be accessed here:


